# Resection of the Urethral Plate and Augmented Ventral Buccal Graft in Patients with Long Obliterative Urethral Strictures

**DOI:** 10.1590/S1677-5538.IBJU.2015.0065

**Published:** 2015

**Authors:** Ivan Ignjatovic, Milan Potic, Dragoslav Basic, Ljubomir Dinic, Darko Laketic, Marija Mihajlovic, Aleksandar Skakic

**Affiliations:** 1 Clinical Center Nis - Clinic of Urology, School of Medicine, Nis, Serbia

## Abstract

The treatment of long urethral strictures is based on the use of buccal mucosa graft (BMG). Postoperative failures commonly occur in patients with the obliterative strictures, and the long augmented part of the urethra which is prone to fibrotic changes.

Combined approach with the resection of the obliterative part of the urethral plate located in the bulbar urethra, together with the ventral placement of BMG was performed in 36 patients. Etiology of the stricture was: idiopathic in 19/36 (52.7%), iatrogenic in 14/36 (38.8%), and other causes in 3/36 (8.3%). Mean length of the stricture was 7.2±1.6 cm, and the length of the augmented graft 4.5±1.2 cm (due to resected urethral plate) so, the single BMG was enough in 25/36 (69.4%) patients. The medium postoperative follow up was 24 months (20–28 months) months. Success of the surgery was defined as no need for additional surgery neither dilatation. Cystoscopy was performed 4–6 months after the surgery and additional follow up with IPSS and uroflowmetry.

Overall success was achieved in 31/36 (86.1%) patients. Mean postoperative IPSS was 9.5±2.1 in these patients. Complications were according to Clavien Dindo scale: grade II in 11/36 (30.5%-infection, orchialgia, scrotal pain), grade III in 4/36 (11.1%-fistula) and grade IV in 5/36 (14.5% - restenosis). Postoperative Q_max_= 13.2±1.2 ml/s. Bell shaped curve was present in 14/36(38.8%).

Our results suggest that overall success rate is similar to the expected values for BMG surgery, and the number of the grafts used is lower due to reduced stricture length.

## ARTICLE INFO

**Available at:** http://www.brazjurol.com.br/videos/november_december_2015/Ignjatovic_1234_1235video.htm

**Int Braz J urol. 2015; 41 (video #9): 1234-5**
