# Supplementary material for: Resection of the Urethral Plate and Augmented Ventral Buccal Graft in Patients with Long Obliterative Urethral Strictures
Source: Int Braz J Urol. 2015 Nov-Dec;41(6):1234–5. doi: 10.1590/S1677-5538.IBJU.2015.0065 (PMC4756954; doi:10.1590/S1677-5538.IBJU.2015.0065)
Supplement: Related texts

# Letter: EDITORIAL COMMENT

**Authors:** Miroslav L. Djordjevic

**Affiliations:** School of Medicine, University of belgrade, Serbia

The surgical treatment of urethral stricture diseases is continually evolving. In recent years there has been continuous discussion with regard to the etiology, location, length, and management of extensive urethral stricture disease. Various tissues such as genital and extragenital skin, buccal mucosa, lingual mucosa, small intestinal submucosa, and bladder mucosa have been proposed for urethral reconstruction (1). Although various surgical techniques are available for the treatment of long anterior urethral stricture, no one technique has been identified as the method of choice. Basically, in patients with a wide, soft urethral plate and no fibrous spongiosum tissue, use of a graft is preferred. Contrary, in patients with a narrow, rigid urethral plate and fibrous spongiosum tissue, use of a flap is preferred. Although a buccal mucosa seems to be better than a skin graft, the difference in success rate is so slight (82% vs. 78%) that it does not justify the use of a buccal mucosa as a first choice (2). In this Video, the authors presented one of the procedures for long urethral stricture. Scientific and technical demonstration of their procedure looks excellent. The authors are to be congratulated on this complex and precize work.

## References

[1] Djordjevic ML (2014). Graft surgery in extensive urethral stricture disease. Curr Urol Rep.

[2] Barbagli G, Morgia G, Lazzeri M (2008). Retrospective outcome analysis of one-stage penile urethroplasty using a flap or graft in a homogeneous series of patients. BJU Int.
